# Supplementary material for: Emergence of task-related spatiotemporal population dynamics in transplanted neurons
Source: Nat Commun. 2023 Nov 11;14:7320. doi: 10.1038/s41467-023-43081-w (PMC10640594; doi:10.1038/s41467-023-43081-w)
Supplement: Supplementary file 1 — Supplementary Information [file 41467_2023_43081_MOESM1_ESM.pdf]

SUPPLEMENTARY INFORMATION

Figure S1

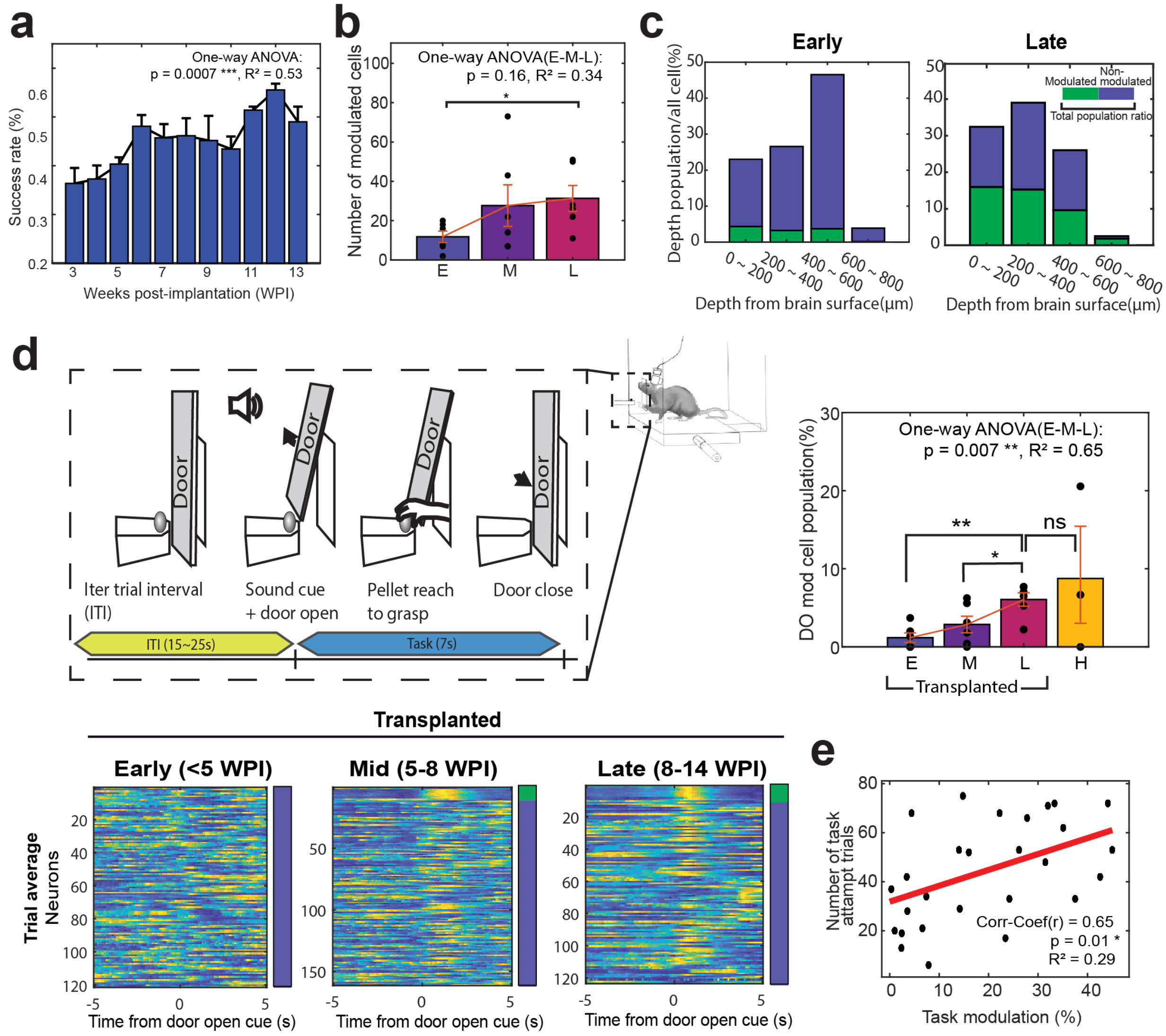

Behavioral performance and task-related neural dynamics in transplanted mice. (a) Weekly behavioral recovery from transplanted mice shows significant behavioral improvements over the weeks (One-way ANOVA, p:0.0007, R^2:0.53). Source data are provided as a Source Data file. (b) Similar to the proportions of task-modulated neurons, total number of task-modulated neurons also shows increases over time (One-way ANOVA, p:0.16, R^2:0.34, t-test: ns $p > 0.05$, and * $p \leq 0.05$, one tailed paired t-test in E-M-L, n=6 transplanted mice). Source data are provided as a Source Data file. (c) Histogram showing cortical depth-dependent distribution of transplanted neurons (n=6 mice, early and late training). While 400-600 μm depth showed the highest number of active neurons at early, all depths displayed similar proportions of task-modulated neurons at both early and late periods. Source data are provided as a Source Data file. (d) Activity from a subset of transplanted neurons became increasingly aligned to door open cue tone (Cue-modulated cell proportions; E: 1.2%, M: 2.8%, L: 6%; One-way ANOVA: p=0.007, R^2=0.65; t-test: ns $p > 0.05$,

* $p \leq 0.05$, and ** $p \leq 0.01$ one tailed paired t-test in E-M-L and one tailed unpaired t-test between L and H), with peak cue-modulation similar to intact motor cortical circuitry. All data are presented as the mean ± SEM. This might be expected since the anterior and medial region surrounding the transplanted graft consists of host pre-motor cortex. Source data are provided as a Source Data file. (e) Task-related activity in transplanted neurons is correlated to number of task-attempt trials (LME: (fixed) $r = 0.65$, $p = 0.01$, $R^2 = 0.29$; (random) intercept std = 4.6, r-std = 7.1×10^{-11}), with high task engagement leading to enhanced task-locked activity. Source data are provided as a Source Data file.

Figure S2

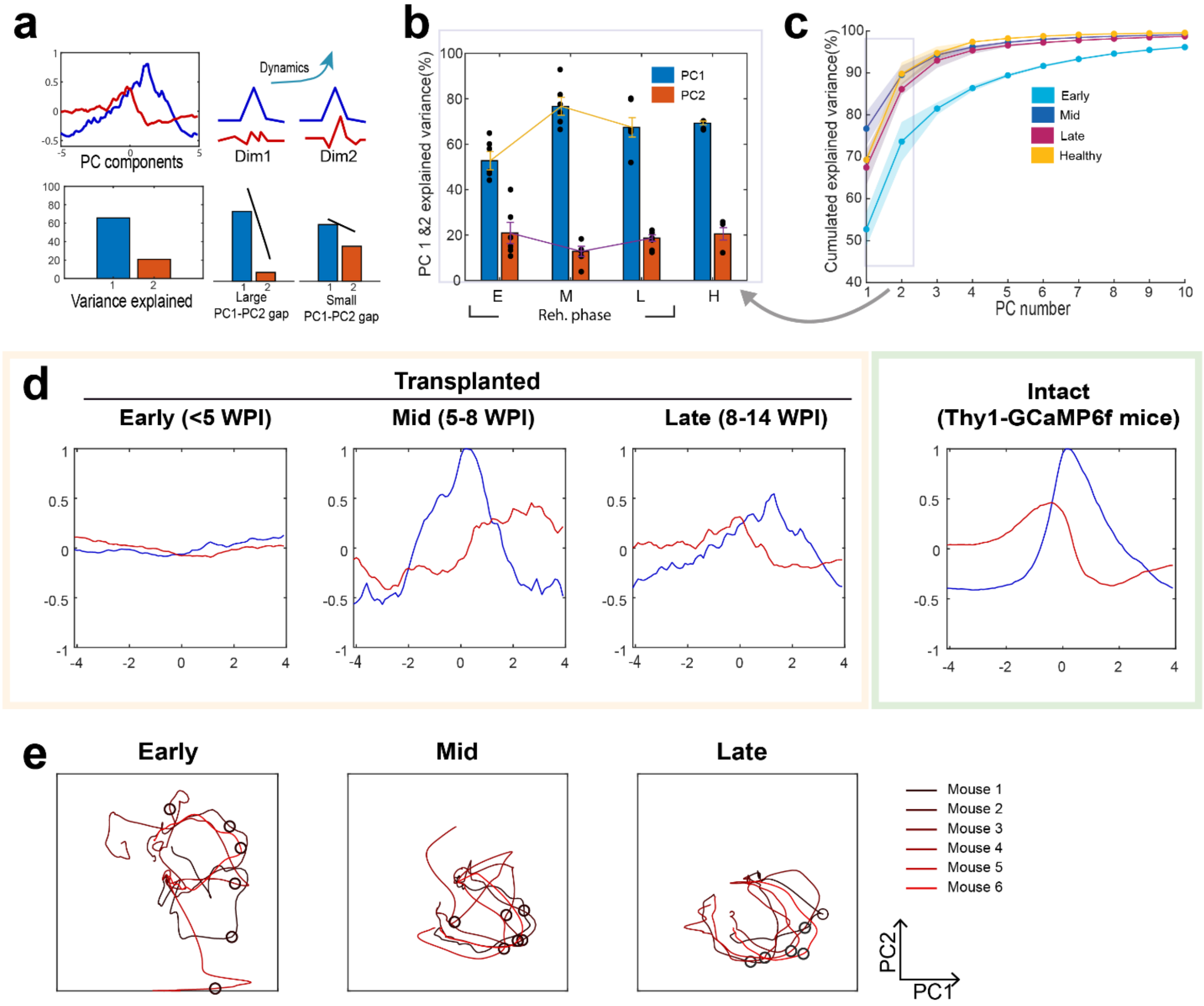

Task-related population dynamics in transplanted neurons. (a) To quantify changes in task dynamics, we also performed PCA to calculate the variance accounted for each PC during the movement epochs. (b) This analysis gives an indication for how much of the neural variance is captured by each PC and suggested that the during late training, variance explained by PC1 and PC2 were similar to variance displayed in healthy mice (n=6 transplanted mice, n=3 healthy mice). All data are presented as the mean ± SEM. Source data are provided as a Source Data file. (c) Both of these components combined explained ~90% of the variance. (d) PC1 dominated during mid sessions, while late sessions were characterized by improved PC1 and PC2 balance, similar to intact circuitry. (e) Rotational dynamics from each of the transplanted mice gradually improved over the weeks. n=6 transplanted mice.

Figure S3

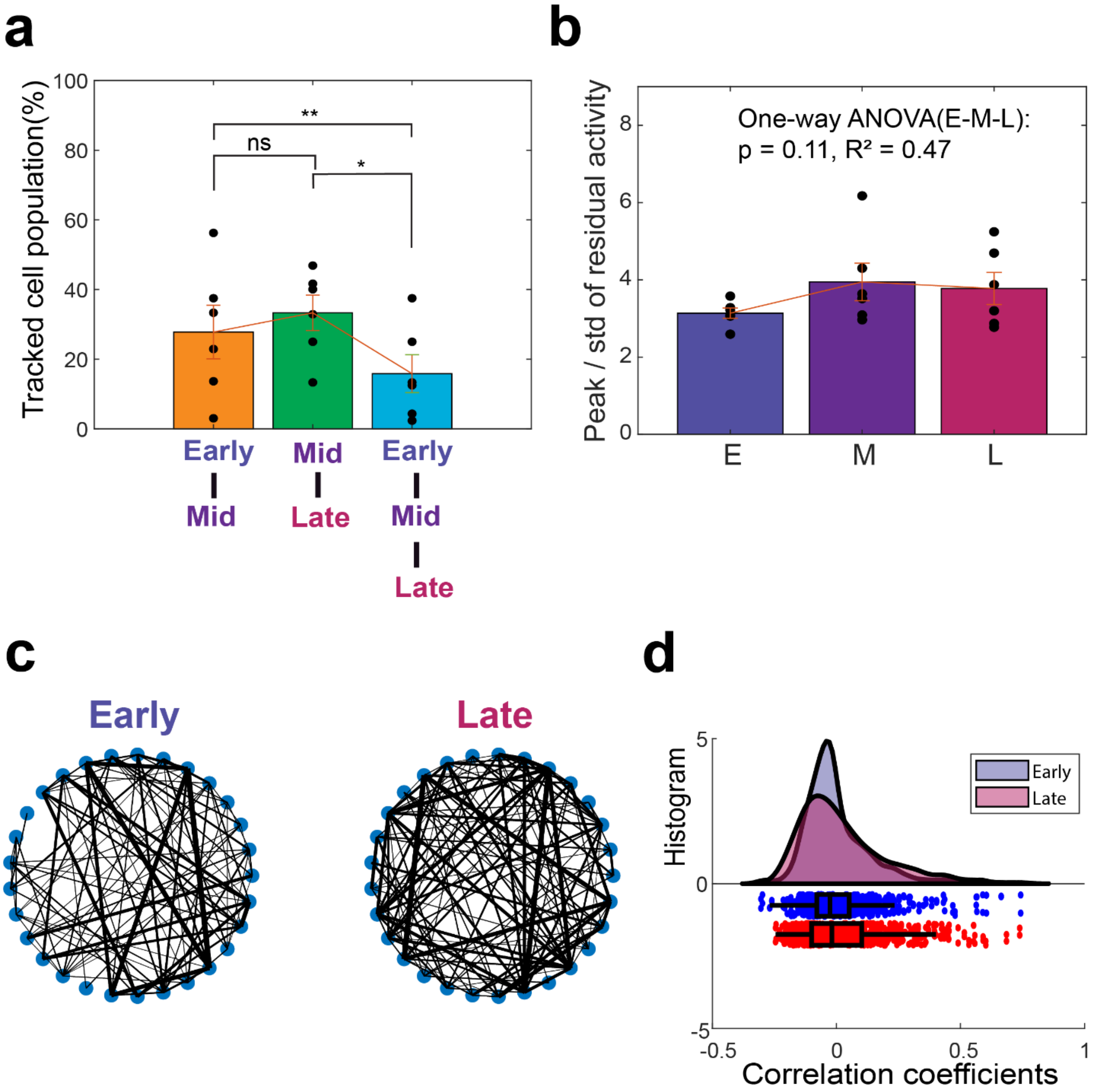

Task-related neural dynamics in tracked neurons. (a) A major challenge in quantifying emergence and stability of neural dynamics is lack of recording techniques that can sample a stable set of neurons over long periods, especially in deep cortical layers. To overcome this limitation, we utilized an implantable PRISM lens and image registration tools (with manual confirmation) to track ~15% of single transplanted neurons across the cortical depth and training period. Cell tracking over a shorter time period (Early-Mid or Mid-Late) yielded significantly higher proportions (~30%) of tracked cell population. One tailed paired t-test: ns $p > 0.05$, * $p \leq 0.05$, and *** $p \leq 0.001$. All data are presented as the mean ± SEM with n=6 transplanted mice. Source data provided as a Source Data file. (b) During the same period, the average calcium peak fluorescence

did not show significant changes (one-way ANOVA: $p = 0.11$, $R^2 = 0.47$; t-test: ns $p > 0.05$, one tailed paired t-test in E-M-L, n=6 transplanted mice). All data are presented as the mean ± SEM. Source data are provided as a Source Data file. (c) Tracked neurons from healthy motor cortical circuitry during motor training yielded similar increases in neural co-variations, (d) including broadening of the distribution due to increase in correlated (both positive and negative) activity. The histogram box plot displays median and percentile boundary (i.e., 25th and 75th at the box edges) and whiskers are 1.5 interquartile range. n=1056 tracked cell correlation from 1 healthy mice. Source data are provided as a Source Data file.

Figure S4

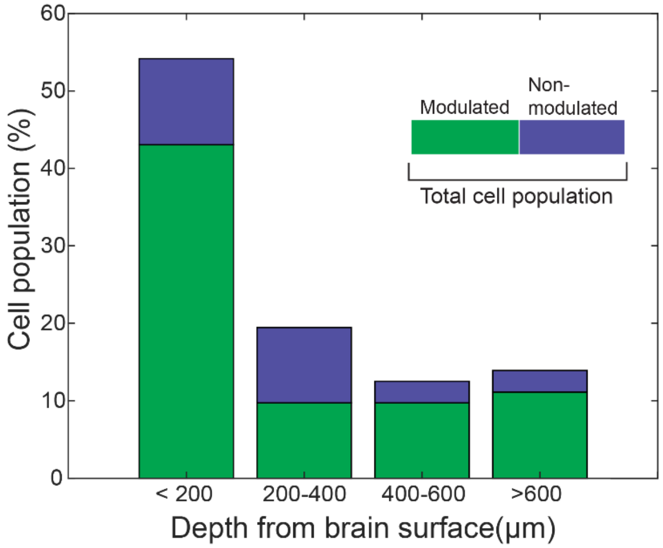

Histogram showing cortical depth-dependent distribution of transplanted neurons from stimulation mice (n=3). Although there are more cells present in upper cortical layers in ACS-treated mice, all depths displayed strong modulation during ACS. Source data are provided as a Source Data file.

Figure S5

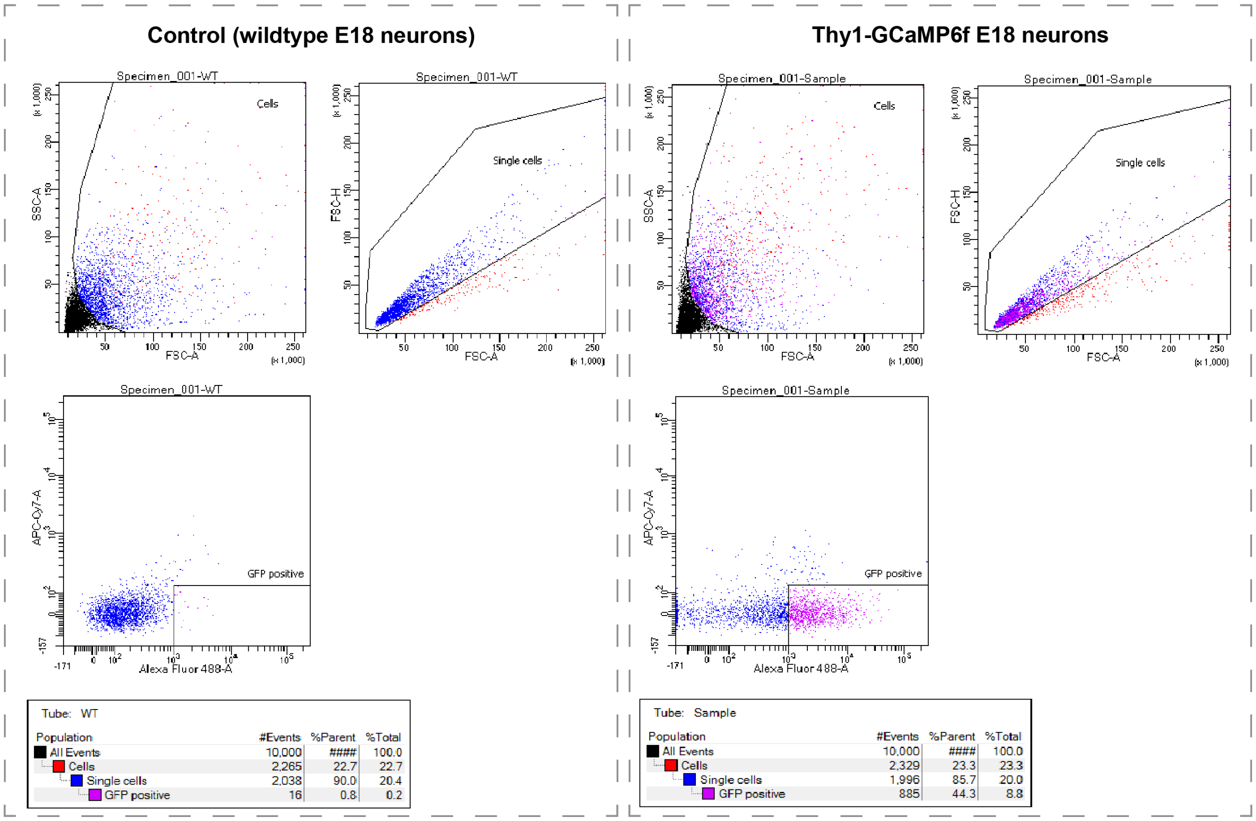

Fluorescence-activated cell sorting (FACS) can be used to isolate distinct cell types expressing calcium indicators for transplantation experiments. For example, here we show that live cortical E18 excitatory pyramidal neurons expressing GCaMP6f under Thy1 promoter can be selectively detected based on baseline GFP fluorescence (without antibody application) and isolated for cell-type specific experiments. The FACS-sorted neurons can be further added to existing cell populations (with or without glia) to manipulate the proportions of excitatory and inhibitory cell-types in the transplanted network. Wildtype (C57BL/6J) E18 cortical neurons show minimal GFP fluorescence and can be used as control mice for cell sorting.

Figure S6

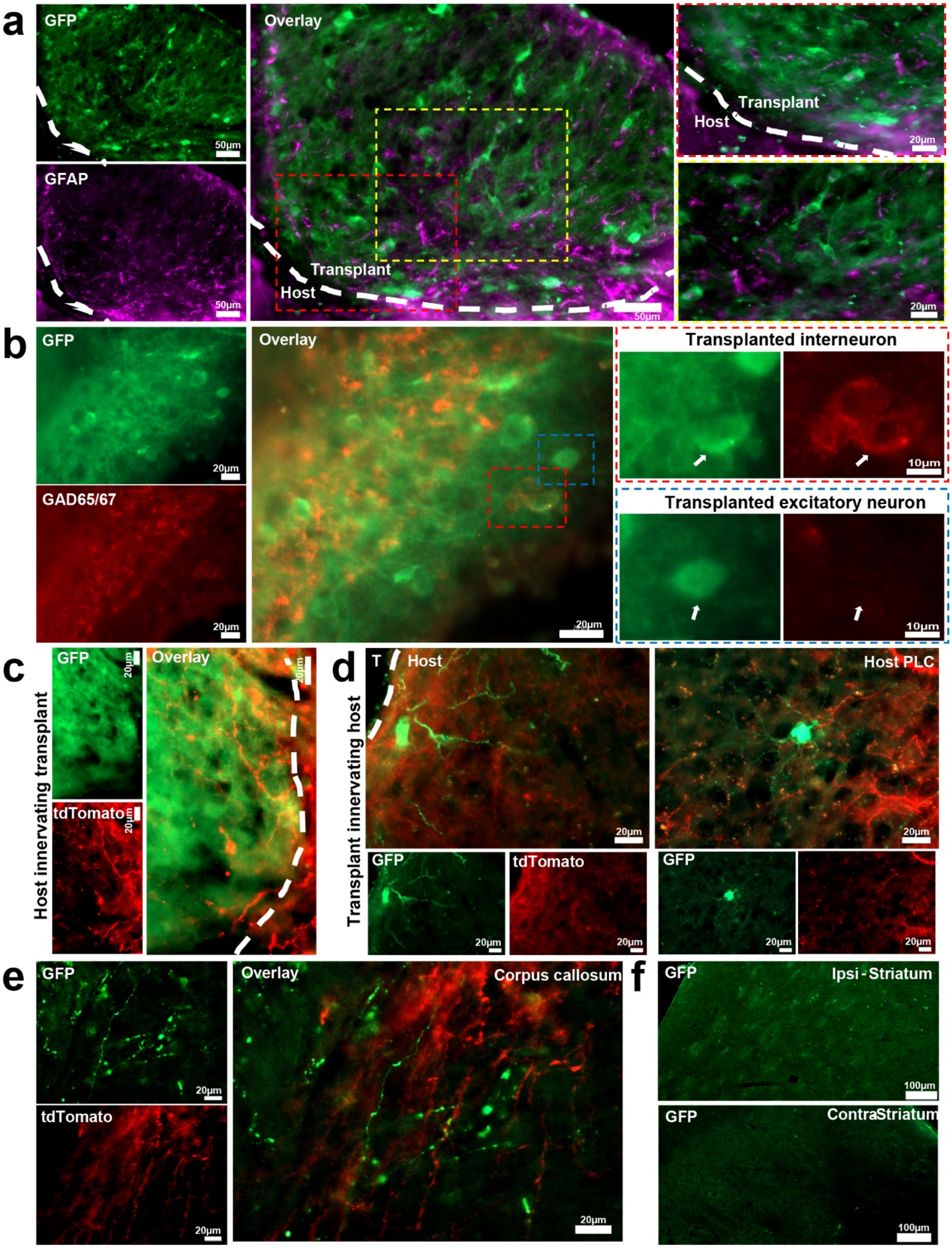
a
GFP
GFAP
Overlay
Transplant
Host
50µm
20µm
b
GFP
GAD65/67
Overlay
Transplanted interneuron
Transplanted excitatory neuron
20µm
10µm
c
Host innervating transplant
GFP
tdTomato
Overlay
20µm
d
Transplant innervating host
T
Host
Host PLC
GFP
tdTomato
20µm
e
GFP
tdTomato
Overlay
Corpus callosum
20µm
f
GFP
Ipsi - Striatum
ContraStriatum
100µm

Histological evaluation of the transplanted graft using fluorescence microscopy. (a) Anti-GFP staining allowed for the identification of transplanted neurons, while the boundary between the host and the stroke cavity (containing the grafted cells) could be identified with anti-GFAP staining. GFAP+ cells could be seen around the lesion boundary, as well as inside the graft. Scale bar: 50 µm, inlets: 20 µm. (b) Staining with anti-GAD65/67, a pan-interneuron marker, revealed that a subset of GFP+ transplanted neurons are inhibitory neurons (GFP+, GAD65/67+), while others showed no labeling (GFP+, GAD65/67-), suggesting excitatory nature of these neurons. Scale bar: 20 µm. inlets: 10 µm. (c) Labeling of host cortical neurons prior to stroke and transplantation revealed substantial infiltration of host axons, labeled with tdTomato, into the GFP+ transplanted graft. Scale bar: 20 µm. (d) While majority of the transplanted neurons stayed within the stroke cavity at 14 WPI, sparse migration into the peri-lesional cortex was evident. Scale bar: 20 µm. (e) In addition to the presence of GFP+ axons in the peri-lesional cortex, we observed axonal projections from transplanted neurons within the white matter tracts, alongside host axons. Scale bar: 20 µm. (f) Sub-cortical labeling was evident in ipsilateral striatum showcasing their ability to extend processes into deeper brain regions. Conversely, the contralateral striatum remained void of any observable labeling, emphasizing the specificity of this sub-cortical axonal projections. Scale bar: 100 µm. Histological images are representative from n=6 transplanted mice.
