# Supplementary material for: Emergence of task-related spatiotemporal population dynamics in transplanted neurons
Source: Nat Commun. 2023 Nov 11;14:7320. doi: 10.1038/s41467-023-43081-w (PMC10640594; doi:10.1038/s41467-023-43081-w)
Supplement: Supplementary file 3 — Description of Additional Supplementary Files [file 41467_2023_43081_MOESM3_ESM.pdf]

Description of Additional Supplementary Files

Supplementary Movie 1: Calcium imaging of transplanted neurons across cortical mantle (600µm x 800 µm). Example video shows 40s of calcium data acquired at 10Hz.

Supplementary Movie 2: Task-related activation of transplanted neurons.

Supplementary Movie 3: Modulation of transplanted neurons using ACS.

Supplementary Movie 4: Blood flow imaging. Example video shows 10s of raw data acquired at 60Hz.
