# Supplementary material for: Emergence of task-related spatiotemporal population dynamics in transplanted neurons
Source: Nat Commun. 2023 Nov 11;14:7320. doi: 10.1038/s41467-023-43081-w (PMC10640594; doi:10.1038/s41467-023-43081-w)
Supplement: Supplementary file 8 — Reporting Summary [file 41467_2023_43081_MOESM8_ESM.pdf]

nature portfolio

Corresponding author(s): Karunesh Ganguly

Last updated by author(s): Oct 15, 2023

Reporting Summary

Nature Portfolio wishes to improve the reproducibility of the work that we publish. This form provides structure for consistency and transparency in reporting. For further information on Nature Portfolio policies, see our Editorial Policies and the Editorial Policy Checklist.

Statistics

For all statistical analyses, confirm that the following items are present in the figure legend, table legend, main text, or Methods section.

n/a	Confirmed	
☐	☒	The exact sample size (*n*) for each experimental group/condition, given as a discrete number and unit of measurement
☐	☒	A statement on whether measurements were taken from distinct samples or whether the same sample was measured repeatedly
☐	☒	The statistical test(s) used AND whether they are one- or two-sided<br>*Only common tests should be described solely by name; describe more complex techniques in the Methods section.*
☐	☒	A description of all covariates tested
☐	☒	A description of any assumptions or corrections, such as tests of normality and adjustment for multiple comparisons
☐	☒	A full description of the statistical parameters including central tendency (e.g. means) or other basic estimates (e.g. regression coefficient) AND variation (e.g. standard deviation) or associated estimates of uncertainty (e.g. confidence intervals)
☐	☒	For null hypothesis testing, the test statistic (e.g. *F*, *t*, *r*) with confidence intervals, effect sizes, degrees of freedom and *P* value noted<br>*Give P values as exact values whenever suitable.*
☒	☐	For Bayesian analysis, information on the choice of priors and Markov chain Monte Carlo settings
☒	☐	For hierarchical and complex designs, identification of the appropriate level for tests and full reporting of outcomes
☒	☐	Estimates of effect sizes (e.g. Cohen's *d*, Pearson's *r*), indicating how they were calculated

Our web collection on statistics for biologists contains articles on many of the points above.

Software and code

Policy information about availability of computer code

Data collection	We collected the data with Inscopix miniscope with Inscopix data acquisition software (IDAS).<br><br>IDAS (Inscopix, Version: v2.0.0, https://www.inscopix.com/software-analysis-miniscope-imaging).
Data analysis	The collected data were first imported to Inscopix data processing software (IDPS) and the data were exported in TIFF format. In python, Caiman performed image movement correction, demixing, denoising, and detrending. The post-processed data were imported to MATLAB for analysis. We made customized code for the analysis (i.e., Modulated cell detection, microcirculatory dynamic analysis etc.).<br><br>IDPS (Inscopix, Version: v1.8.0, https://www.inscopix.com/software-analysis-miniscope-imaging)<br>Python (Python Software Foundation, Version: v3.8.13, https://www.python.org)<br>Matlab (Mathworks, Version: R2020b, https://www.mathworks.com/products/matlab.html)<br>Caiman (Flatiron Institute, Version: v1.9.8, https://github.com/flatironinstitute/CaImAn)<br><br>Custom MATLAB codes for modulated cell detection and microcirculatory dynamics are available at https://github.com/gangulylab/CellTPCode.

For manuscripts utilizing custom algorithms or software that are central to the research but not yet described in published literature, software must be made available to editors and reviewers. We strongly encourage code deposition in a community repository (e.g. GitHub). See the Nature Portfolio guidelines for submitting code & software for further information.

Data

Policy information about availability of data

All manuscripts must include a data availability statement. This statement should provide the following information, where applicable:

- Accession codes, unique identifiers, or web links for publicly available datasets
- A description of any restrictions on data availability
- For clinical datasets or third party data, please ensure that the statement adheres to our policy

Source data are provided as a Source Data file. The calcium imaging data at Early, Mid, and Late is used in this study have been deposited at 10.5281/zenodo.10003677

Research involving human participants, their data, or biological material

Policy information about studies with human participants or human data. See also policy information about sex, gender (identity/presentation), and sexual orientation and race, ethnicity and racism.

Reporting on sex and gender	N/A
Reporting on race, ethnicity, or other socially relevant groupings	N/A
Population characteristics	N/A
Recruitment	N/A
Ethics oversight	N/A

Note that full information on the approval of the study protocol must also be provided in the manuscript.

Field-specific reporting

Please select the one below that is the best fit for your research. If you are not sure, read the appropriate sections before making your selection.

- [x] Life sciences
- [ ] Behavioural & social sciences
- [ ] Ecological, evolutionary & environmental sciences

For a reference copy of the document with all sections, see nature.com/documents/nr-reporting-summary-flat.pdf

Life sciences study design

All studies must disclose on these points even when the disclosure is negative.

Sample size	No statistical methods were used to predetermine sample sizes, but our sample sizes are similar to those reported in previous studies analyzing neural dynamics in motor cortex (Kim JK et al 2023, Arroyo S et al 2023). We used 3 mice for healthy controls since healthy adults display highly stereotypes movements and stable behavioral performance (and neural sequencing) during reach to grasp task. We used 6 adult stroke mice for transplant experiments to account for the behavioral and neural variability post-stroke.
Data exclusions	Detected components with poor, low quality signals (SNR < 2) were excluded during CalmAn processing of the calcium data. After processing, detected components were then manually excluded based on the shape of the component (i.e. irregular shapes unlike soma) and so that there was minimal overlap between detected cells. Sessions with less than 20% task attempt rate were also excluded from analysis due to low number of trials.
Replication	All relevant behavioral experiments were independently performed in all 9 mice (6 stroke and 3 healthy) and neural effects were present in all animals. Data shown is representative of the results across all mice. Effects reported were consistent across animals and significant at the appropriate group level.
Randomization	Since there is only one treatment group, randomization was not performed. However, each stroke mouse was randomly assigned to receiving either Syn-GCaMP or CamK-GCaMP embryonic neurons.
Blinding	Blinding between the treatment and healthy groups was not relevant for these experiments since long-term (3 months) tracking of neural population and behavioral training was only performed for stroke mice receiving the cells, while healthy mice typically reach stable performance in < 10 days.

Reporting for specific materials, systems and methods

We require information from authors about some types of materials, experimental systems and methods used in many studies. Here, indicate whether each material, system or method listed is relevant to your study. If you are not sure if a list item applies to your research, read the appropriate section before selecting a response.

Materials & experimental systems

n/a	Involved in the study
	☒ Antibodies
☒	☐ Eukaryotic cell lines
☒	☐ Palaeontology and archaeology
	☒ Animals and other organisms
☒	☐ Clinical data
☒	☐ Dual use research of concern
☒	☐ Plants

Methods

n/a	Involved in the study
☒	☐ ChIP-seq
	☒ Flow cytometry
☒	☐ MRI-based neuroimaging

Antibodies

Antibodies used	mouse anti-GFP (Abcam #ab1218), rabbit anti-GFAP (Abcam #7260), rabbit anti-GAD65/67 (Abcam #ab183999). Information about working dilutions used are listed in the methods section.
Validation	These antibodies have been validated by the manufacturer for mouse brain tissues. Before testing these antibodies on transplanted cells, we further validated these antibodies on healthy mouse brain tissue.

Animals and other research organisms

Policy information about studies involving animals; ARRIVE guidelines recommended for reporting animal research, and Sex and Gender in Research

Laboratory animals	We used six wildtype C57BL/6J (Jackson Laboratory, Jax #000664) 10-12 weeks old adult male mice as hosts for stroke and transplantation experiments. For isolating donor embryonic cortical neurons expressing GCaMP6f, we crossed 10-12 weeks old female Ai148D GCaMP6f reporter mice (Jax #030328) with pan-neuronal60,61, Syn1-Cre (Jax #003966, n=3) and CaMKIIα-Cre (Jax #005359, n=3) male mice. While we used male host mice for current experiments as a proof-of-principle to show dynamic integration of transplanted neurons, future experiments will involve mice from both sex. We also used 10-12 weeks old male Thy1-GCaMP6f (Jax #025393, n=3) as control mice to study network dynamics in healthy cortical networks.
Wild animals	No wild animals were used in this study.
Reporting on sex	Wildtype and Thy1 males were used for for implantation and behavioral experiments.
Field-collected samples	No field samples were collected in this study.
Ethics oversight	Experiments were approved by the Institutional Animal Care and Use Committee at the San Francisco VA Medical Center.

Note that full information on the approval of the study protocol must also be provided in the manuscript.

Flow Cytometry

Plots

Confirm that:

☒ The axis labels state the marker and fluorochrome used (e.g. CD4-FITC).

☒ The axis scales are clearly visible. Include numbers along axes only for bottom left plot of group (a 'group' is an analysis of identical markers).

☒ All plots are contour plots with outliers or pseudocolor plots.

☒ A numerical value for number of cells or percentage (with statistics) is provided.

Methodology

Sample preparation	E18 Thy1-GCaMP6f neurons were isolated using same preparation methods as transplantation experiments. Once isolated, cells were then processed for flow cytometry to identify GFP+ cell populations. No antibody was applied on live cells to enhance GFP signal.
Instrument	BD FACS Aria II
Software	BD FACSDiva 8.0.1
Cell population abundance	8.8% of total events processed were identified as GFP+ neurons in the Thy1 cortices, while wildtype cortices were determined to be 0.2% of total events.
Gating strategy	Similar gating strategy was used for wildtype (GFP-) and Thy1-GCaMP (GFP+) cell populations so that most of the cells (99.8%)

from wildtype cortices were considered to be in the GFP negative boundary. Exact details on gating boundary are shown in supplementary figure 5.

☒ Tick this box to confirm that a figure exemplifying the gating strategy is provided in the Supplementary Information.
